# Cystitis, Acute

**Published:** 1883-11

**Authors:** Charles L. Swift


### CYSTITIS, ACUTE.

*Read before the Cayuga County Homœopathic Medical Society, June 14th, 1883.*

CHARLES L. SWIFT, M. D.

Symptoms of inflammation of the bladder are excessive frequency in passing water, attended with much pain, also pain about the loins, pelvis, and perineum; tenderness to pressure over the pubic region and in the rectum. The urine may contain one or all of the following—mucus, pus, blood, and lymph—the blood increasing as the disease advances. There is considerable fever, which is marked by frequent rigors, followed by heat and sweat.

The causes of cystitis are various, such as cold, strains, injuries, prolonged labors, retention of urine, gonorrhœa, astringent injections, and extension of inflammation from the kidneys, ureters, or from the uterus.

The treatment of cystitis is of two kinds, allopathic or palliative, and homœopathic or remedial, "specific."

Here is good old orthodox treatment, palliative: First examine the patient, and if there is retention of urine, introduce a catheter and draw it off; if this does not relieve, apply hot fomentations to the supra-pubic region; hot hip baths; must have an action of the bowels; must give dilutent drinks; frequent small doses of Alkali; full doses of Henbane (Hyosciamus), ten to twenty drops of fluid extract; small doses of Morphia or Chlorodyne; suppositories of Morphia or Opium when the pain is very severe.

In mild cases the patient may take three or four ounces of the infusion of Buchu three times a day, or a pint of the strong decoction of Triticum repens daily.

If these fail to restore health, what then?—they must die, or call on specific treatment.

Let me report two or three cases:

CASE I.—Mr. S., aged 40, light complexion, laborer, was taken with a severe chill, followed by severe pain in the region of the bladder, frequent urination, and finally retention. Urine was drawn off with a catheter and was very dark and bloody, after which he could pass it.

He was under the care of one of our allopathic physicians for two weeks. He grew worse, passed nothing but blood, and so weak that they thought he would never be any better.

His son came to me and asked me to send some medicine to his father. He gave me these symptoms, with those already given: Great burning before, during, and after micturition, with great urging, passing but little at a time; great thirst, but drink made him feel worse, increasing the pain in the bladder; he felt better when keeping quiet. Gave him Canth.[30] to be put in water, and repeated every hour until he began to feel better, then not so frequent. The next day the blood was absent from the urine, less burning, and on the third day was able to walk out-doors. In this case Morphine and Opium failed to relieve the patient, while a very few pellets of Canth. did not only relieve, but cured the case.

Case II.—Mrs. C., aged 50, took a severe cold, followed by a chill and fever. The next morning, after the chill, awoke with severe pains through the bladder, back, hips, and down the thighs. Frequent urination, passing but little, and that mostly blood, with a great deal of burning and straining.

This condition lasted three days before seeking medical relief, she then sending for some medicine. Sent her Canth. Next day was called to see her. Found her worse instead of better.

Here was something wrong. This was certainly a case of cystitis, or the books are wrong. Canth. cured one case of cystitis. Should it not cure all if it is a specific? Must I give Morphine and Opium, Henbane and Alkali, to cure it? We will examine our patient better, now we see her. She has all the symptoms given—frequent urination, but little at a time, with burning and very severe pain; but the pain was at the conclusion of passing urine, when it was almost unbearable. Gave this time, not Canth., but Sarsaparilla[12], which cured as promptly as Canth. in the first case.

It is not the name of the disease we cure, but the symptoms, and if we cure these we cure the patient.

Case III.—Mrs. O., aged 27, had the following symptoms: Pain in the supra-pubic region, back, and hips; tenderness all over the hypogastric region and in the rectum; constipation, defecation painful; passes urine every few minutes, with burning sensation, as if she had held her water too long. Has to rise at night to urinate; urine smells very badly; cannot allow it to remain in the room; sediment sticks to the vessel and has to be washed off. Chilly by spells; icy-cold feet. Nervous and fidgety.

Was this a case of anteversion of the uterus? or was it cystitis? or both, or neither? Gave Sepia[30], and cured the case.

From these and other cases which I might give, I ask these questions: How are we to treat this disease, unless we treat it by the symptoms? Is it necessary to diagnose the case to cure it? Is it necessary to call in an allopathic doctor to help you tell what is the matter? or will you carefully examine the patient and get a complete history of the case and all of its symptoms, and give the remedy indicated by the symptoms and cure your case? Which will you do?

